# Model calculations for neutron-induced reactions in meteorites and planetary surfaces

**DOI:** 10.1186/s40623-025-02358-8

**Published:** 2026-01-14

**Authors:** Ingo Leya

**Affiliations:** Space Science and Planetology, University of Bern, 3012, Bern , Switzerland

**Keywords:** Galactic cosmic rays, Production of secondary neutrons, Neutron-induced reactions, Geant4, Thermal neutrons

## Abstract

This study investigates the capabilities of the GEANT4 Monte Carlo toolkit to quantitatively predict neutron production, neutron transport, and nuclide production by neutron capture reactions in cosmochemical relevant objects. The model reproduces neutron densities measured in the lunar surface within the experimental uncertainties, which is a major improvement compared to earlier studies. Since, for many applications in meteorites and planetary surfaces, nuclide production by neutron capture is of importance, the production of $^{41}$Ca and $^{60}$Co is studied as an example. In addition, shifts in the stable isotope ratios $^{157}$Gd/$^{160}$Gd, $^{158}$Gd/$^{160}$Gd, $^{149}$Sm/$^{152}$Sm, and $^{150}$Sm/$^{152}$Sm (and combinations thereof) are modeled and compared to experimental data. The model describes experimental $^{41}$Ca activity concentrations in different types of meteorites and the lunar surface within the uncertainties. In contrast, it fails to describe $^{60}$Co activity concentrations. In addition, it is difficult to consistently model the isotope shifts $^{157}$Gd/$^{160}$Gd and $^{150}$Sm/$^{152}$Sm in Apollo 15 drill core samples. The observed trends depend on the temperature of the irradiated object and are more pronounced for colder temperatures. Since the observed discrepancies are likely related to the shape of the neutron spectra, self-shielding effects by, e.g., $^{56}$Fe, might be of importance and some of the consequences are discussed.

**Graphical Abstract:**

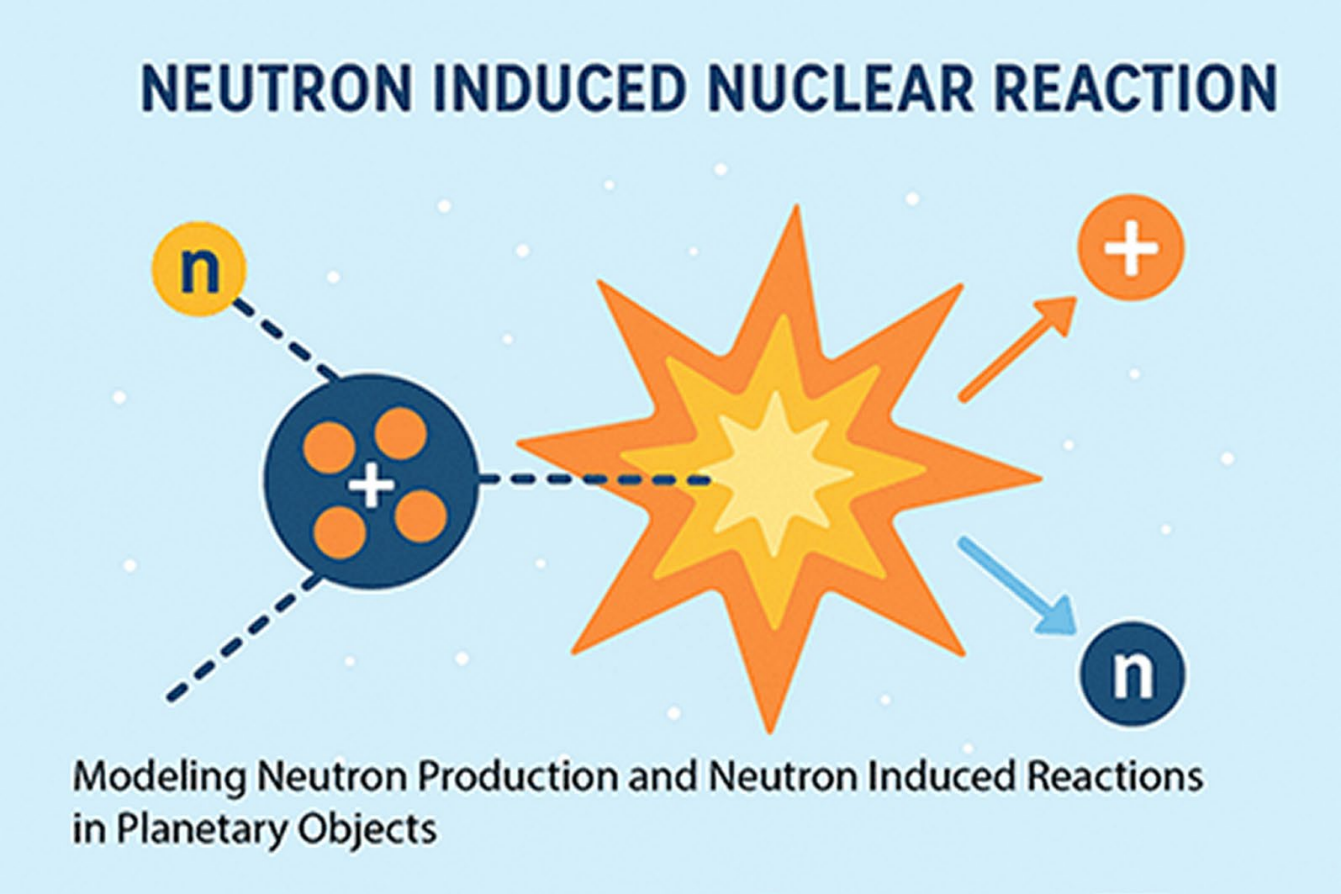

## Introduction

Cosmic ray interactions with meteorites, planetary surfaces, and atmospheres generate secondary particles—primarily neutrons but also protons, light charged particles, and occasionally more exotic species such as muons and kaons—in a process known as a cosmic ray-induced cascade. The nuclear products formed by reactions of both primary and secondary cosmic ray particles, known as cosmogenic nuclides, are widely used to reconstruct and characterize the irradiation histories of the materials being studied (e.g., Masarik and Reedy 1994, 1996a; Wieler 2002; Ammon et al. 2009; David and Leya 2019; Leya et al. 2021) but also to understand the history of the cosmic rays itself (e.g., Wieler et al. 2013; Smith et al. 2019; Leya et al. 2021; Poluianov and Engelbrecht 2025).

While most of the established cosmogenic nuclides, e.g., $^{10}$Be, $^{26}$Al, $^{36}$Cl, $^{41}$Ca, $^{53}$Mn, $^{60}$Fe, and the stable noble gas isotopes, are produced by relatively high energetic projectiles, i.e., by protons and neutrons with energies above a few MeV, the highest particle flux in meteorites and the lunar surface are typically for neutrons in the energy range below $\sim$1 MeV. For terrestrial in situ studies, neutrons are even the sole contributors to cosmogenic nuclide production (at least directly at the surface, in deeper layers muon-induced reactions are also of importance).

Since some of the Rare Earth Elements (REE), e.g., Sm and Gd, have relatively large capture cross sections for such low energy neutrons, they can produce measurable isotopic shifts that were used to study the gardening history of regolith, e.g., for the Moon and the aubrite parent body. In addition, such shifts have been used to study the irradiation histories of Martian meteorites, chondrites, mesosiderites, and iron meteorites. For references, see (e.g., Leya and Masarik 2013). Prominent examples are isotopic shifts in $^{157}$Gd/$^{160}$Gd, $^{158}$Gd/$^{160}$Gd, $^{149}$Sm/$^{152}$Sm, and $^{150}$Sm/$^{152}$Sm (and combinations thereof) in meteorites and lunar samples (e.g., Hidaka et al. 2000, 2011, 2020).

Neutron capture effects sometimes produce a nuisance for stable and radioactive nuclide studies. Examples are the Hf-W, Mn-Cr, and Pd-Ag dating systems used to establish an early solar system chronology. In such studies, uncorrected cosmogenic effects from neutron capture can significantly alter the determined ages and often introduce substantial scatter in the datasets. For a recent study using the long-lived dating system $^{176}$Lu-$^{176}$Hf that needed to be corrected for cosmogenic effects on Hf isotopes, see Dauphas et al. (2025).

In addition, neutron and gamma spectroscopy of cosmic ray-induced signals using remote sensing techniques have been utilized in various space missions to study, among others, the elemental composition of the Moon, Mars, and the asteroids Vesta and Eros. In contrast to optical spectroscopy techniques, neutron and gamma spectroscopy can also study the subsurface layers (depending on the neutron energy up to 1 m deep) and can therefore give crucial information not otherwise attainable. For some references, see (e.g., Lawrence et al. 2002; McKinney et al. 2006; Ota et al. 2011; Prettyman et al. 2013; Mesick et al. 2018).

A dedicated study modeling thermal and epithermal neutron capture effects in radioactive and stable nuclide systems has been published by Leya and Masarik (2013). In this study, the neutron spectra were calculated using the Los Alamos High Energy Transport (LAHET) code system. In the newest model for cosmogenic production rates in meteorites and planetary surfaces (Leya et al. 2021; Leya 2025), the primary and secondary particle spectra are calculated using the GEANT4 (GEometry And Tracking) toolkit [Agostinelli et al. (2003); Allison et al. (2016)] using version 11.2.2 (https://geant4.org/download/11.2.2.html). In view of the recent impressive advances and improvements in GEANT4 for modeling nuclear reactions and particle transport, it seems appropriate and necessary to revisit and reevaluate the model calculations for neutron capture effects in meteorites, planetary surfaces, and planetary atmospheres. For modeling cosmogenic nuclide production in terrestrial and extraterrestrial materials by thermal and/or epithermal neutron capture reactions, the irradiated targets are usually mixtures of different elements, most of them arranged in certain minerals. The targets therefore differ from the targets typically considered for model evaluations in more technical driven applications, for which pure metals and/or relatively simple molecules are sufficient. This study aims at evaluating the model predictions for more complex targets relevant for geo- and cosmochemical applications.

## Modeling particle spectra and nuclide production with Geant4

For most Monte Carlo codes, neutron transport is distinguished into three different regimes, depending on energy. For high energies, i.e., energies above a few hundred keV, neutrons see the target nuclei as fixed points without any motion, because the kinetic energy of the traveling neutrons is so much larger than the thermal energy of the target nuclei. This energy range is usually labeled *fast spectrum*. For energies below a few hundred keV but greater than a few eV, the energy range called *epithermal*, the traveling neutrons see the target as a gas of nuclei in thermal equilibrium with a Maxwellian velocity distribution corresponding to the temperature of the target. In this energy domain, nuclear reactions between the traveling neutron and the target nuclei occur at a relative velocity and are described by the so-called free gas approximation. At lower energies, i.e., in the *thermal energy* domain, the neutron energies and therefore the neutron wavelengths are comparable to the chemical bonding energies of the target. As a consequence, the neutron is not interacting with individual nuclei but with the target material as a whole. In the Monte Carlo codes, taking into account molecular/material effects is via the so-called thermal scattering law (TSL), which is available in evaluated nuclear data libraries like ENDF/B (e.g., Chadwick 2011; Brown 2018) or JEFF (Plomben et al. 2020). The TSL is particularly important for material and/or crystalline lattices of low-mass materials, because the energy transfer between the neutron and the target molecules is maximized for such targets. Unfortunately, thermal scattering data are only available for a few materials. For other materials, free gas scattering models are used, i.e., the material properties are not taken into consideration. For more information, see, e.g., Tran et al. (2018); Thulliez et al. (2022).

For modeling particle spectra inside planetary bodies and/or inside technical installations like particle detectors and neutron spallation sources, physics models following particle production and particle transport are needed. One of the most versatile and probably best developed models is the GEANT4 Monte Carlo toolkit (Agostinelli et al. 2003; Allison et al. 2016), which has been used before modeling cosmogenic production rates in meteorites and the lunar surface (e.g., Mesick et al. 2018; Leya et al. 2021; Čechvala et al. 2023; Feige et al. 2024; Tauseef et al. 2024; Ghaznavi et al. 2025; Leya 2025).

In a recent study, Tran et al. (2018) compared the thermal neutron scattering treatment between MCNP6 and GEANT4 and found that there is general agreement for most materials (less than 5% deviations). However, for some relevant materials, such as Fe and Al as metals, there are still some disagreements between the codes of 21% and 11%, respectively. Note that the thermal neutron physics in MCNP6 and GEANT4 is not the same and therefore differences are expected. More recently, Thulliez et al. (2022) compared the predictions of GEANT4 with the results obtained using the Monte Carlo neutron transport code TRIPOLI-4, which has been developed exclusively to study shielding, reactor physics, and fusion systems. This code is benchmarked regularly and is therefore considered as a reference. The differences between the two codes GEANT4 and TRIPOLI-4 were in the range of $\sim$2%, which confirms the accuracy of the GEANT4 neutron treatment.

### The *PhysicsList* in Geant4

In GEANT4, the user can choose a PhysicsList, which specifies which particles will exist and be tracked in the simulation, assigns processes to the particles (e.g., ionization, scattering, decay), selects out of the many different models for the same physical interaction the one that is most suitable for the particular application, and also sets important parameters such as cut-off parameters that determine when certain interactions or decays are no longer simulated. For the hadronic interactions important for the present study, the following models are relevant. At energies greater than $\sim$ 20 GeV, there is a choice between the Fritiof string model (FTF, above $\sim$5 GeV) or the Quark-Gluon String model (QGS, above $\sim$20 GeV). The FTF model handles hadron-nucleus collisions at energies above 3–4 GeV/c with an upper limit of applicability of about 1000 GeV/c. The QGS physics list also uses a string model approach to simulate inelastic interactions of hadrons above $\sim$15–20 GeV. Both models can be used with or without considering a precompound part (P), which is an extension of the hadronic models toward lower energies and should provide a smooth transition between the different stages of the model, especially between the kinetic models describing the first stage of the reaction and the models describing the final de-excitation of the (hot) nucleus during the final equilibrium part of the reaction.

The intranuclear cascade (INC) can be modeled using BERTINI, INCL++, or BIC. The cascade begins when an incident particle strikes a nucleon inside the target nucleus and transfers some of its energy in an inelastic collision. In this way, further nucleons are excited. and some of them can leave the nucleus, predominately in forward direction. After some collisions, the cascade ends when all particles, which are kinematically able to leave the nucleus, have escaped. After this step, a complete energy equilibration is assumed and further energy loss is via isotropic emission of particles (including $\alpha$ particles). In the BERTINI model, there is a pre-equilibrium step between the INC phase and the evaporation phase. After renewed interest in spallation reactions, INCL++ combined with an evaporation model has been developed and significantly improved (e.g.,Cugnon et al. 2007; Boudard et al. 2013; David 2015). INCL++ is currently the most reliable spallation model available (e.g., David 2015; David and Leya 2019; David et al. 2008, 2011; Leray et al. 2011). The supported energy range of INCL++ for incident nucleons and pions is 1 MeV–20 GeV and any target nucleus is (in principle) possible. In addition to protons, neutrons, pions, and kaons, light nuclei from A = 2 to A = 18 can also be used as projectiles. INCL++ is also able to simulate the emission of light composite particles up to A=8 during the cascade stage using a coalescence algorithm for cluster formation.

As with all INC models, INCL++ simulates only the first part of the spallation reaction; the de-excitation of the excited nucleus after the cascade is treated by a dedicated de-excitation code. The Binary Cascade model (BIC) is a new approach to cascade calculations as it mixes the approaches of quantum molecular dynamics with generalized cascade models, i.e., BIC uses a 3-dimensional model of the nucleus and exclusively considers binary scattering between reaction participants and the nucleons within the (3d) nuclear model. It is therefore a hybrid between the classical cascade models (BERTINI, INCL++) and quantum molecular dynamic models. BIC has a wide range of applicability in nucleon-nuclear reactions ranging from less than 100 MeV up to $\sim$10 GeV. For more information, see, e.g., Folger et al. (2004). For recent improvements and performance tests of the model, see, e.g., Apostolakis et al. (2009).

For many cosmochemical applications, an accurate description of the interactions between low energetic neutrons (*E* < 20 MeV) and the irradiated material is of fundamental importance. GEANT4 uses optional neutron data files of high precision neutron models and includes elastic and inelastic scattering, absorption, and fission. For using the neutron high precision model, the HP package must be activated. The model HPT is almost identical to HP except that a special treatment of elastic scattering of thermal neutrons is activated.

The PhysicsList is simply assembled by adding the different names of the modules. For example, using the Fritiof string model (FTF) with the pre-equilibrium part (P) in combination with the INCL++ intranuclear cascade phase (INCXX) gives the PhysicsList: FTFP$_{-}$INCLXX. Adding then the HP package for the neutrons gives the final PhysicsList: FTFP$_{-}$INCLXX$_{-}$HP.

In the present study, the irradiation of a lunar drill core was calculated using the following eight PhysicsLists: FTFP$_{-}$INCLXX$_{-}$HP, FTFP$_{-}$INCLXX$_{-}$HPT, FTFP$_{-}$BERT$_{-}$HP, FTFP$_{-}$BERT$_{-}$HPT, QGSP$_{-}$INCLXX$_{-}$HP, QGSP$_{-}$INCLXX$_{-}$HPT, QGSP$_{-}$BIC$_{-}$HP, QGSP$_{-}$BIC$_{-}$HPT. In addition, the two PhysicsLists QGSP$_{-}$BIC$_{-}$ALLHPT and QGSP$_{-}$BIC$_{-}$ALLHP were used, which are identical to QGSP$_{-}$BIC$_{-}$HP except that for protons and light ions the ParticleHP module is used, i.e., the modeling is based on evaluated databases. Finally, the PhysicsList QBBC, which is recommended for medical and space applications, is used. Therefore, in total, 11 different PhysicsLists were studied. More information can be found in the GEANT4 Guide for PhysicLists (Release 11.2, 2023).

### The Primary galactic cosmic ray source

One of the most important input data for any model of cosmogenic nuclide production is the primary spectrum of galactic cosmic ray (GCR) particles inside the solar heliosphere. Various parameterizations exist for describing the GCR spectrum in the solar system. All of them are derived from a local interstellar spectrum (LIS) and considering solar modulation effects using the force-field approximation (e.g., Gleeson and Axford 1967, 1968), which is a simplified version of the original transport equation, and which assumes azimuthal and spherical symmetry. In this approach, the energy spectrum of the *i*th GCR species in the inner heliosphere is related to the unmodulated LIS of the same species through a solar modulation potential *M* (in MV).

1 $$\begin{aligned} J_{{\mathrm{GCR}},i}(E,t) = J_{{\mathrm{LIS}},i}(E+M(t)) \times \frac{E(E+m_p c^2)}{(E+M(t))\cdot(E+M(t)+2m_p c^2)} \end{aligned}$$

where *E* is the kinetic energy per nucleon. The solar modulation potential *M*(*t*) varies with time. For cosmogenic nuclide studies in terrestrial and extraterrestrial samples, the parameterization originally developed by Castagnoli and Lal (1980) and later revised by Masarik and Reedy (1996b) is widely used:

2 $$\begin{aligned} J_{{\mathrm{GCR}}} = c_p = \frac{E(E + 2m_p c^2) \cdot (E + x + M(t))^{-2.65}}{(E + M(t)) \cdot (E + 2m_p c^2 + M(t))} \end{aligned}$$

where *E* is the kinetic energy in MeV, $m_p$ the proton rest mass, and the factor *x* is given by:

3 $$\begin{aligned} x = 780 \times e^{-2.5 \cdot 10^{-4} \times E} \end{aligned}$$

The constant $c_p = 9.9 \times 10^8$ ensures that the flux has units of particles m$^{-2}$ s$^{-1}$ sr$^{-1}$ MeV$^{-1}$.

As discussed in detail by Leya et al. (2021), this is the first version of the model that directly includes both primary and secondary galactic $\alpha$-particles. Earlier models relied on a relatively crude approximation in which production rates were scaled from calculations that considered only primary protons along with secondary protons and secondary neutrons. To make the model calculations more convenient, we developed a parameterized representation of the primary galactic $\alpha$-particle spectrum. This parameterization is based on the assumption that the primary $\alpha$-particle spectrum is very similar to the primary proton spectrum when both are expressed as functions of energy per nucleon. With this approach, particle spectra in meteorites, planetary atmospheres, and planetary surfaces can be calculated using only a single value of the solar modulation potential. For further details, see Leya et al. (2021). It is important to note that the discussion below, specifically the treatment of the solar modulation potential, is only valid for spectra expressed in the format of Castagnoli and Lal (1980).

### Solar modulation

Using the Castagnoli-Lal spectrum (index *CL*), the long-term, i.e., in the range of a few mîllion years, average solar modulation potentials ($M_{{\mathrm{CL}}}$) at 1 AU and in the meteoroid orbits at 3-5 AU were determined to be 660 MV and 600 MV, respectively (Leya et al. 2021). However, this long-term average is not directly applicable to the solar modulation at the time of the Apollo missions. Using the spectral shape for primary GCR particles as proposed by Usoskin et al. (2005) (index *U*), the studies by Mesick et al. (2018) and Usoskin et al. (2005) give a solar modulation potential $M_{U}$ = 532 MV with a typical error of 35–40 MV for the year 1972, i.e., for the year the Apollo astronauts performed the Lunar Neutron Probe Experiment (LNPE, see below). However, this value is not applicable to the present study because the primary GCR spectrum given by Usoskin et al. (2005) differs from the Castagnoli-Lal local interstellar spectrum. It is important to mention that the specific value of the solar modulation potential *M* is attributed to a specific LIS. However, it is possible to recalculate the *M* value for one LIS to the corresponding *M* value of another LIS. As shown by Usoskin et al. (2005) and Leya et al. (2021), there is an (almost) linear relationship. For the present study, the modulation $M_{U} = 532$ MV valid for the spectrum by Usoskin et al. (2005) corresponds to a solar modulation $M_{{\mathrm{CL}}} = 438$ MV for the spectrum given by Castagnoli and Lal (1980). Consequently, when evaluating the model calculations using the Apollo LNPE data and/or short-lived nuclides measured in Apollo samples, particle spectra calculated using a spectrum given by Castagnoli and Lal (1980) with a solar modulation $M_{{\mathrm{CL}}} = 438$ MV must be used. If, in contrast, long-lived or stable nuclides are discussed, a solar modulation potential $M_{{\mathrm{CL}}} = 660$ MV must be used (for the same primary GCR spectrum).

### The irradiated targets

For modeling the particle spectra for the lunar LNPE experiment performed during the Apollo 17 mission, the following chemical composition is used for the regolith (weight percent): O (42.06), Na (0.3145), Mg (6.11), Al (6.755), Si (19.47), K (0.1017), Ca (7.734), Ti (4.16), Cr (0.279), Mn (0.163), Fe (12.87), Sm (0.000848), Eu (0.000171), Gd (0.001123), and Th (0.0001534) (Laul et al. 1978, 1979). The density is $\rho$ = 1.86 g/cm^3^ (Carrier 1974; Mitchell et al. 1973). For more information, see also McKinney et al. (2006) and Mesick et al. (2018). For the GEANT4 calculations, the surface of the Moon is divided into 100 layers, each 2.5 cm thick. The covered depth of 250 cm therefore corresponds to 465 g/cm^2^. For modeling, a homogeneous target composition is assumed. Though, calculations using inhomogeneous samples are planned.

The H5 chondrite Jilin is modeled as a spherical body with a radius of 85 cm, which has been divided into 34 concentric shells, each with a thickness of 2.5 cm. The chemical composition used for modeling is (weight percent): C (0.11), O (34.14), Na (0.66), Mg (14.42), Al (1.16), Si (17.59), P (0.12), S (2.04), K (0.08), Ca (1.28), Ti (0.07), Mn (0.250), Fe (28.20), Co (0.08), Ni (1.79), U (0.000358), and Th (0.000149) (e.g., Wang and Rubin 1987). A density of 3.40 g/cm^3^ is used. However, one must take the irradiation record of Jilin into accoung. Initial analyzes of cosmogenic noble gases and radionuclides indicated a two-stage exposure history; a first irradiation stage on a very large object with a radius of more than 10 m for $\sim$10 Ma and a second 4$\pi$ irradiation stage as a spherical object with a radius of 85 cm for $\sim$4 Ma (Honda et al. 1982; Heusser et al. 1985; Pal et al. 1985). In a later study, the irradiation history was revised to 7 Ma for the first exposure stage and 0.32 Ma for the second exposure stage (Heusser et al. 1996).

In a follow-up study, thermal neutron capture effects will be modeled for the production of $^{36}$Cl from $^{35}$Cl, $^{41}$Ca from $^{40}$Ca, and $^{129}$I from $^{128}$Te as well as neutron-induced isotopic shifts in some relevant stable nuclides (W. Pd). These calculations will be carried out for ordinary chondrites, HED meteorites, ureilites, carbonaceous chondrites, martian meteorites, iron meteorites, and the lunar surface. The results will be presented in a separate publication. Details of the targets and results for the production of long-lived radionuclides are already available in Leya (2025).

For modeling, the temperature can be specified, by default it is 273.15 K. Since moderation and build-up of thermal neutrons depend on the temperature of the irradiated object, systematic tests at different temperatures in the range 100–500 K were performed.

### Modeling nuclide production

For modeling nuclide production in meteorites (see below), the following equation is used:

4 $$\begin{aligned} P_{i}(\vec {r},\vec {c}_{{\mathrm{sample}}},\vec {C}_{{\mathrm{meteoroid}}},R) = \sum _{k} \sum _{j} \vec {c}_{j} \int _{E} \sigma _{i,j,k}(E) \phi _{k}(\vec {r},\vec {C}_{{\mathrm{meteoroid}}},R,E) dE \end{aligned}$$

where $P_i$ (atoms/g/s) is the production rate of nuclide *i* in a meteoroid of radius *R* (cm) at position $\vec {r}$. The chemical composition of the studied sample is $\vec {c}_{{\mathrm{sample}}}$ (1/g) and $\vec {C}_{{\mathrm{meteoroid}}}$ (1/g) is the chemical composition of the entire meteoroid. The differential flux density of primary and secondary particles of type *k* is $\phi _{k}(\vec {r},\vec {C}_{{\mathrm{meteoroid}}},R,E)$ in (1/s/cm^2^/MeV), which depends on the pre-atmospheric geometry, the depth of the sample within the pre-atmospheric meteoroid, and the bulk chemical composition of the meteoroid. The nuclear cross section for the production of nuclide *i* from chemical element *j* by particles of type *k* is $\sigma _{i,j,k}(E)$ (cm^2^), and $\vec {c}_{j}$ represents the concentration of element *j* in the studied sample (assumed to be constant) (1/g). Note that the chemical composition of the studied sample $\vec {c}_{j}$ must not be the same as the chemical composition of the entire meteoroid $\vec {C}_{{\mathrm{meteoroid}}}$, if, for example, silicate inclusions in an iron meteoroid are investigated. For simplicity it is usually assumed that the pre-atmospheric meteoroid was spherical and that the cosmic ray flux was temporally constant. For the present study, *k* = Neutrons, because only reactions induced by secondary neutrons are considered.

The cross sections for the production of $^{60}$Co from $^{59}$Co and $^{41}$Ca from $^{40}$Ca were taken from the ENDFB-VIII database (Brown 2018). To check the robustness of our results, we also performed calculations using cross sections from the JEFF database and from earlier versions of the ENDFB database. The calculated production rates show almost no dependence on the choice of the neutron cross section database

For the numerical calculation, the step size of the integration is the same as the step size of the neutron capture cross sections. Since the energy resolution of the neutron capture cross sections is much higher than that for the particle spectra, this set-up guarantees that all structures and resonances are fully considered. For the integration, which is from $10^{-12}$ MeV to $10^4$ MeV, Simpsons rule is used.

## Results

This section presents some results and findings relevant for neutron capture studies in terrestrial and extraterrestrial material. First, the influence of the target temperature (as given in GEANT4) on the neutron density and on the shape of the neutron spectra is discussed. Second, the influence of the GEANT4 physics list on the neutron density is studied, and third, the dependence of the neutron density on the solar modulation potential is modeled. Furthermore, activity concentrations of $^{41}$Ca and $^{60}$Co measured in well studied meteorites and lunar surface samples are compared to the model predictions. Finally, isotope shifts in $^{157}$Gd/$^{160}$Gd, $^{158}$Gd/$^{160}$Gd, $^{149}$Sm/$^{152}$Sm, and $^{150}$Sm/$^{152}$Sm (and combinations thereof) are investigated. These comparisons enable to draw important conclusions regarding the applicability of the model to geo- and cosmochemical applications.

### The influence of the temperature on the particle spectra

Since this study focuses on thermal neutrons and thermal neutron-induced nuclear reactions, an investigation of whether the temperature of the target affects the modeled neutron spectra and therefore the production rates is necessary. The reasoning is a follows, at low energies, the neutron wavelength becomes comparable to the chemical bonding energies and therefore the neutron no longer interacts with individual nuclei but with the target material as a whole. Finally, such a neutron—if not being absorbed - reaches thermal equilibrium with its surroundings and the average kinetic energy is similar to the average kinetic energy of the surrounding atoms and/or molecules. Physically speaking, in equilibrium matches the Maxwell-Boltzmann distribution of the neutrons those of the surrounding atoms and/or molecules.

**Fig. 1:**
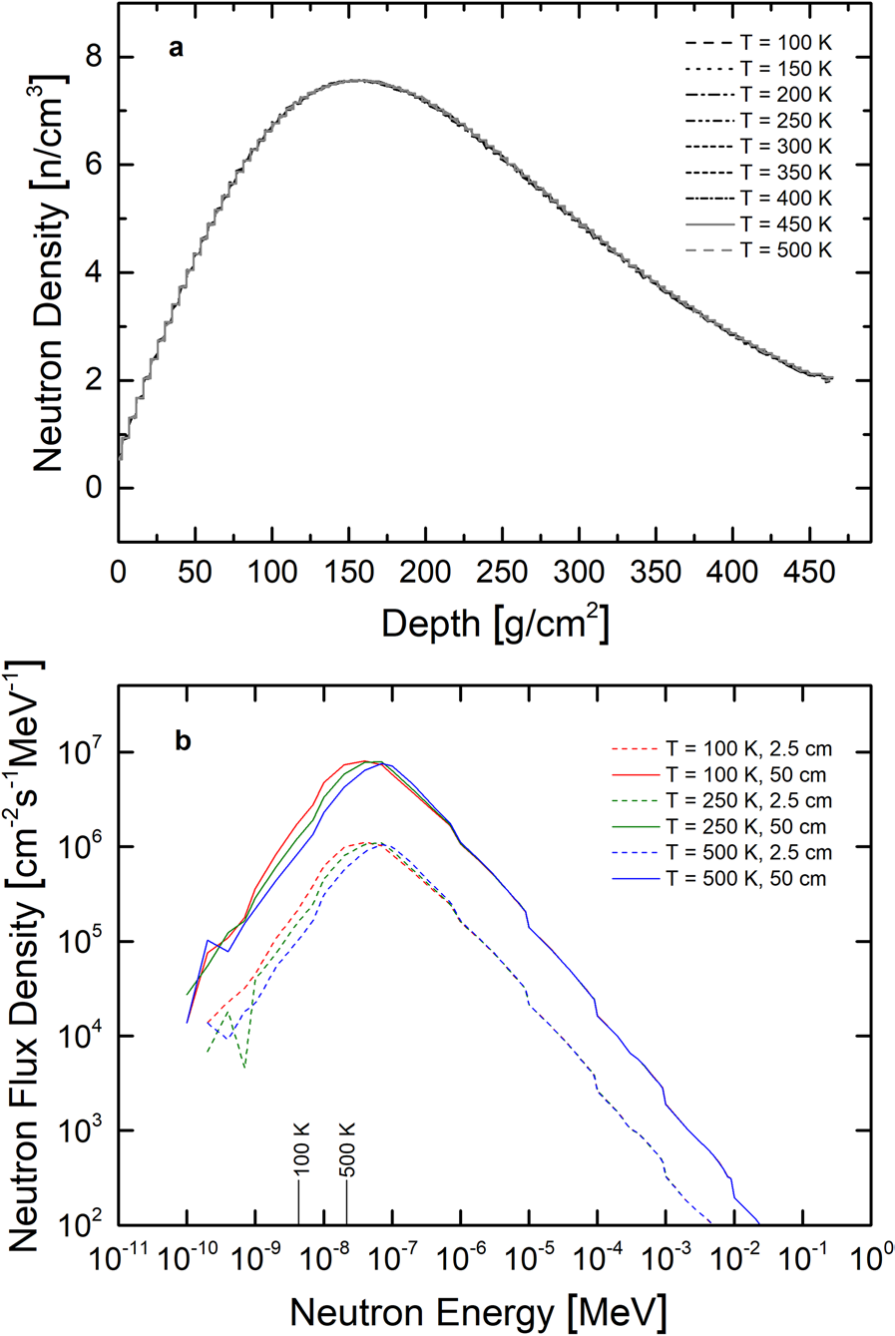
Neutron density vs. depth for different target temperatures (**a**) Neutron spectra for different temperatures (**b**). Panel a shows modeled neutron densities for the LNPE experiment as a function of depth below the surface. For modeling, the target temperature was varied from 100 K to 500 K in steps of 50 K. Panel b depicts the neutron flux density as a function of neutron energy for energies below 1 MeV. Shown are spectra at depths of 2.5 cm and 50 cm for three different target temperatures (100 K, 250 K, 500 K)

The differential neutron flux densities (1/cm^2^/s/MeV) and the neutron densities (1/cm^3^) were modeled for the LNPE drill core for temperatures between 100 K and 500 K in steps of 50 K using the GEANT4 physics list FTFP$_{-}$INCLXX$_{-}$HPT. For a definition of the neutron densities and a discussion of the results from the different physics lists see below. The results for the neutron densities are shown in Fig. 1a. There is no dependency of the neutron density on the temperature, all curves are essentially identical. This finding is expected considering that most of the neutrons are at energies much higher than thermal and a shift of the Maxwell-Boltzmann distribution toward higher or lower values would not affect the neutron density, which is an integrated signal (see below). In contrast, one expects that the neutron spectra at low energies are temperature dependent. This is seen in Fig. 1b, where the spectral shapes at depths of 2.5 cm and 50 cm are shown for irradiations at 100 K, 200 K, and 500 K. Shown are the neutron spectra in the unit (1/cm^2^/s/MeV) in the energy range 10$^{-10}$ MeV to $\sim$10$^{-1}$ MeV. For better interpretation, also the thermal energies corresponding to 100 K and 500 K are indicated on the x-axis. The maximum of the spectra at low energies, which corresponds to the peak of the Maxwell-Boltzmann distribution, shows the expected trend with temperature. The peak for the 100 K irradiation is at lower energies than the peak for the 250 K irradiation, which is at a lower energy than the peak for the 500 K irradiation. The most probable energy for a Maxwell-Boltzmann distribution at 100 K is 4.31 × 10$^{-9}$ MeV, which is a factor of $\sim$10 lower then the position of the peak modeled via GEANT4. For the 500 K irradiation, the most probable energy is 2.15 × 10$^{-8}$ MeV, which is a factor of $\sim$4 lower than the GEANT4 result (8.1 × 10$^{-8}$ MeV). The peak position of 4.6 × 10$^{-8}$ MeV given by GEANT4 for the 100 K irradiation corresponds to a Maxwell-Boltzmann temperature of $\sim$1000 K and the peak position for the 500 K irradiation of 8.1 × 10$^{-8}$ MeV corresponds to a Maxwell-Boltzmann temperature of $\sim$1900 K. Consequently, for the spectra modeled using GEANT4, the peak of the thermal neutron spectra is at higher energies than expected considering the temperature of the irradiation, the differences are larger for lower temperatures. Currently it is not clear why there are such discrepancies, i.e., why GEANT4 predicts Maxwell-Boltzmann distributions at too high temperatures (energies). Further below, it will be discussed whether this discrepancy has any significant effect on modeled production rates.

### The neutron density on the Moon

**Fig. 2:**
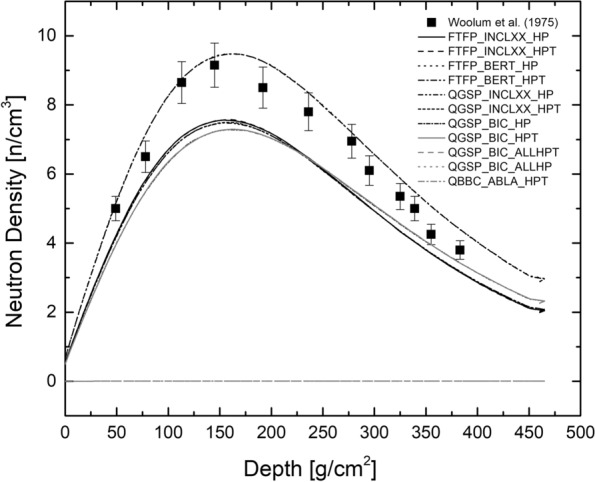
Neutron density vs. depth for different PhysicsLists. Shown are neutron densities for the LNPE experiment as a function of depth below the surface. The experimental data are from Woolum et al. (1975). The model calculations were performed using 11 different PhysicsLists of GEANT4 to study the influence of the PhysicsList on the results

The model was tested by comparing GEANT4 results to the lunar neutron density profile measured by the Apollo 17 experiment. The LNPE experiment measured the neutron density within the first 400 g/cm^2^ of the lunar surface using two types of detector systems, which were inserted into a drill core hole for 49 h and then returned to Earth for analysis. The detectors were sensitive to neutrons with energies less than 500 eV. Details of the experimental set-up and the results can be found by Woolum and Burnett (1974); Burnett and Woolum (1974), and Woolum et al. (1975). The neutron densities, which are given for 12 depths, are shown in Fig. 2 as solid black symbols.

The results of the GEANT4 calculations are particle spectra that can be transferred to neutron densities via:

5 $$\begin{aligned} \rho _{{\mathrm{Neutron}}}(d) = \int _{0}^{500eV} \frac{\Phi _{{\mathrm{Neutron}}}(d,E)}{v_{{\mathrm{Neutron}}}(E)} dE \end{aligned}$$

where $\Phi _{{\mathrm{Neutron}}}(d,E)$ is the flux density at depth *d* (cm$^{-2}$s$^{-1}$MeV$^{-1}$) and $v_{{\mathrm{Neutron}}}(E)$ is the neutron velocity (cm/s). The calculated neutron density has the unit (1/cm^3^) and is therefore directly comparable to the measured data. The results are discussed in the following subsections.

#### Dependence of the neutron density on the PhysicsList used for modeling

Figure 2 depicts the neutron densities calculated via GEANT4 using the 11 PhysicsLists (see above). For modeling, a solar modulation potential $M_{{\mathrm{CL}}}$ = 660 MV is used, though this value is not directly applicable to the data obtained during the Apollo missions (see above). This main purpose of this part of the project is to study if and by how much the choice of the physics list affects the outcome of the modeling. The question of the solar modulation potential is discussed further below. Most of the results are very similar, exceptions are the two physics lists using the Bertini code for the INC phase (FTFP$_{-}$BERT$_{-}$HP and FTFP$_{-}$BERT$_{-}$HPT), which both produce neutron densities that are systematically higher than all the other physics lists. As another exception, the two physics lists QGSP$_{-}$BIC$_{-}$ALLHP and QGSP$_{-}$BIC$_{-}$ALLHPT are failing to produce substantial amounts of neutrons. All other physics lists produce very similar neutron densities, i.e., they are all similar in size and all have a maximum at $\sim$150 g/cm^2^. All further studies are based on the physics lists FTFP$_{-}$INCLXX$_{-}$HP and/or FTFP$_{-}$INCLXX$_{-}$HPT, which produce very similar neutron densities, i.e., the different treatment of thermal neutron elastic scattering is not affecting the results.

Very similar tests have been performed by McKinney et al. (2006) using the MCNPX radiation transport code and by Mesick et al. (2018) using the GEANT4 toolkit, both with mixed results. While the results obtained with MCNPX are in reasonable agreement with the experimental data, there is no information about the primary particle spectrum used for modeling and how this correpond to the used solar modulation potential. In the study by Mesick et al. (2018), the GEANT4 predictions are all significantly higher than the experimental neutron densities. Depending on the chosen physics list, the overestimation was in the range 20-40%. Importantly, the authors concluded that the calculations using INCLXX for the intranuclear cascade phase give the best results. In a study of neutron production in the lunar surface by galactic $\alpha$-particles, Ota et al. (2011) had only limited success modeling the neutron densities, though their approach focussed on finding scaling factors for neutron production between galactic protons and galactic $\alpha$-particles.

#### Dependence of the neutron density on the solar modulation potential

**Fig. 3:**
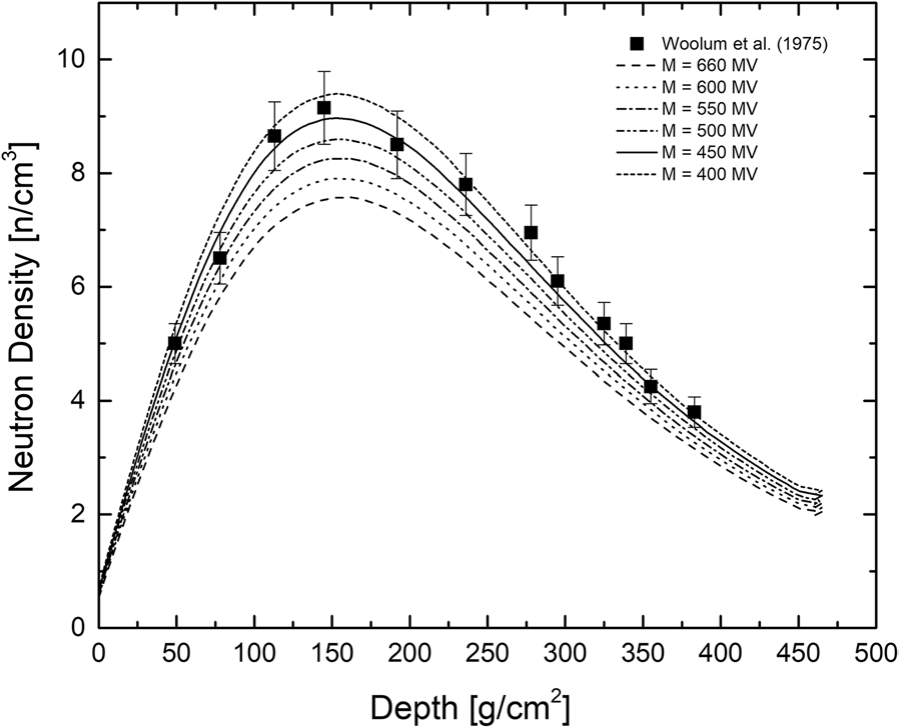
Neutron densities as a function of depth below the surface. For the model calculations, the solar modulation potential was varied between 400 MV and 660 MV. The best agreement between model calculations and experimental data (Woolum et al. 1975) is for a solar modulation potential $M_{{\mathrm{CL}}}$ = 450 MV

Figure 3 compares neutron densities for the LNPE experiment calculated using GEANT4 (PhysicsList FTFP$_{-}$INCLXX$_{-}$HPT) to experimental data, which are from Woolum and Burnett (1974); Burnett and Woolum (1974); Woolum et al. (1975). For modeling, solar modulation potentials $M_{{\mathrm{CL}}}$ = 400, 450, 500, 550, and 600 MV were used. The neutron densities increase with decreasing modulation, i.e., with increasing number of low- and medium-energy primary GCR particles. The best agreement between model predictions and experimental data is for a modulation potential in the range $M_{{\mathrm{CL}}} \sim$ 450 MV, which perfectly agrees with the solar modulation potential of $M_{{\mathrm{CL}}}$ = 438 MV determined above to be valid for the time of the Apollo missions. Therefore, the new version of GEANT4 describes the measured neutron densities within the uncertainties (using the correct solar modulation potential) and therefore performs much better in terms of neutron production and neutron transport than the earlier version of GEANT4 (Mesick et al. 2018) but also compared to MCNPX (McKinney et al. 2006), and PHITS (Ota et al. 2011), at least for neutrons with energies in the range eV to keV as studied by the LNPE experiment. Note that the Apollo missions were during the declining phase of solar activity, which explains the lower solar modulation potential.

To summarize, by using GEANT4 with the physics list FTFP$_{-}$INCLXX$_{-}$HPT and the correct solar modulation potential ($M_{{\mathrm{CL}}}$ = 438 MV), the neutron densities measured during the Apollo mission are reproduced within the uncertainties, indicating the applicability of the model.

### Thermal neutron capture in meteorites and the lunar surface

The neutron densities discussed above are integrated signals, i.e., they correspond to an integral of the neutron spectra over a given energy range that is determined by the experimental set-up. For most geo- and cosmochemical applications, however, the shape of the neutron spectrum is relevant because the shape determines the neutron capture rates and therefore the produced activity concentrations. The latter is the measurable quantity relevant for cosmogenic nuclide studies. In the following, neutron capture effects are studied exemplary for the two relevant cosmogenic nuclides $^{60}$Co, which has a sharp capture resonance at $\sim$0.1 keV, and $^{41}$Ca, for which the cross sections below 1 keV roughly follow a 1/*v*-type shape. The production rates discussed below were calculated using the *conventional* approach by calculating the particle spectra and then folding them with the cross sections of the relevant nuclear reactions (Eq. 1). For comparison, production rates can also be calculated *directly*, i.e., by only using the results from GEANT4. For a discussion and comparisons, see Leya (2025).

#### Thermal neutron capture in meteorites—$^{41}$Ca and $^{60}$Co in Jilin

The Jilin chondrite (H5) is one of the largest stony meteorites recovered so far. The total known weight is about 4 tons and the pre-atmospheric radius has been estimated by the Jilin Consortium Study I (1985) and later by the Jilin Consortium Study II (1996) at 85 ± 8 cm. The two studies provided valuable insights into the cosmic ray exposure history, concluding that Jilin experienced a two-stage exposure history, with a first exposure stage on a very large object with a radius of more than 10 m for about 7 Ma followed by a second $4\pi$-irradiation stage as a spherical body with a radius of 85 ± 8 cm for about 0.32 Ma (e.g., Heusser et al. 1985, 1996; Perron et al. 1996). Due to its large size, Jilin has been extensively studied for neutron capture reactions (e.g., Heusser et al. 1985, 1996; Perron et al. 1996; Hidaka et al. 2011) and therefore provides an excellent benchmark for model calculations.

**Fig. 4:**
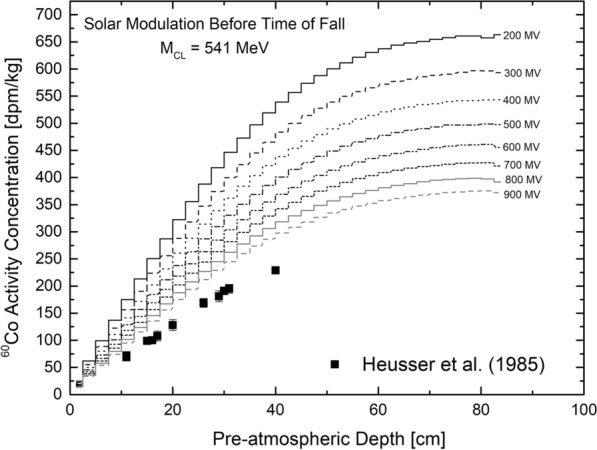
$^{60}$Co activity concentration as a function of depth below the pre-atmospheric surface for the Jilin meteorite. Modeling was performed using solar modulation potentials $M_{{\mathrm{CL}}}$ in the range 200–900 MV. The solar modulation at the time of fall for Jilin was $M_{{\mathrm{CL}}}$ = 541 MV. The experimental data are from Heusser et al. (1985)

Figure 4 depicts measured and modeled $^{60}$Co ($T_{1/2}$ = 5.2714 years) activity concentrations (in disintegrations per minute and kg) for the Jilin chondrite. The experimental data are from Heusser et al. (1985). A Co concentration of 792 ppm is used and the model calculations were performed for solar modulation potentials ranging from $M_{{\mathrm{CL}}}$ = 200 MV to $M_{{\mathrm{CL}}}$ = 900 MV, i.e., for almost the entire range of modulation potentials. The modeled $^{60}$Co depth profiles show a strong dependence on solar modulation; they systematically decrease with increasing solar modulation. This trend is expected because with lower modulation there are more primary GCR particles inside the heliosphere and therefore higher flux densities of secondary neutrons and consequently higher neutron capture rates. All modeled depth profiles are higher than the experimental data, the discrepancies reach a factor of $\sim$2. The reconstruction of solar modulation potentials from Usoskin et al. (2005) gives an average solar modulation potential for the ten years before the fall of the Jilin meteorite of $M_{U} \sim$ 589 MV, which corresponds to $M_{{\mathrm{CL}}} \sim$ 493 MV (see above). However, comparing the experimental data with the modeled depth profile for $M_{{\mathrm{CL}}}$ = 500 MV demonstrates that the model significantly and systematically overestimates the experimental data by $\sim$60%, almost independent on the depth within the meteorite, i.e., there is a constant offset in the model calculations.

For comparison, the $^{60}$Co production rates were also calculated using the direct approach, i.e., by using GEANT4 directly. The thus determined production rates reach values larger than 1000 dpm/kg for a solar modulation potential $M_{{\mathrm{CL}}}$ = 600 MV, i.e., they are even higher than the production rates calculated using the conventional approach. Therefore, the finding that the model overestimates $^{60}$Co production rates holds for the conventional and for the direct approach but is even worse for the direct approach.

This discrepancy cannot be resolved by adjusting the temperature of the irradiation. For example, changing the temperature of Jilin during the irradiation from 10 K to 1500 K changes the $^{60}$Co production rates by less than 13%; the irradiation at 100 K gives the lowest, the irradiation at 1500 K gives the highest production rates. Therefore, while there is a dependency of the production rates on the temperature, it is by far not sufficient to explain the observed discrepancy.

Nishiizumi et al. (1991a) measured in Jilin $^{41}$Ca (T$_{1/2} = 9.94\times 10^4$ years) activity concentrations in the range 200 dpm/kg(Ca) and 2000 dpm/kg(Ca), and argued that the peak concentration is expected to be in the range $\sim$2000 dpm/kg(Ca). Using a solar modulation potential $M_{{\mathrm{CL}}}$ = 600 MV, which is valid for the long-lived radionuclides (Leya et al. 2021), the model predicts $^{41}$Ca activity concentrations of $\sim$70 dpm/kg(Ca) at the surface of Jilin and $\sim$2000 dpm/kg(Ca) at the center, which is in accord with the experimental data. Therefore, the discrepancy seen in $^{60}$Co is not seen in $^{41}$Ca.

#### Neutron capture in meteorites—$^{41}$Ca and $^{60}$Co in Allende

**Fig. 5:**
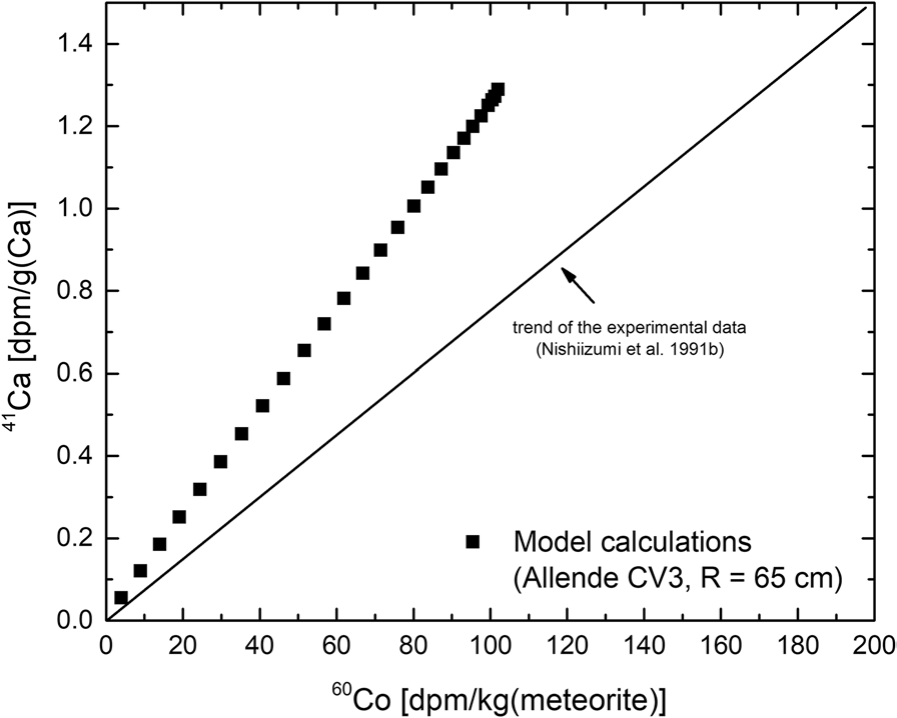
$^{41}$Ca activity concentration as a function of $^{60}$Co activity concentration for the Allende meteorite. The trend given by the experimental data is shown by the solid black line (Nishiizumi et al. (1991b). The results of the model calculations are shown by the black symbols. The different symbols represent different depths in the pre-atmospheric body; increasing concentrations indicate increasing depths

The Allende meteorite is one of the most thoroughly studied carbonaceous chondrites (type CV3). The meteorite fell on February 8, 1969, near the town of Pueblito de Allende in Chihuahua, Mexico. The atmospheric entry was accompanied by a brilliant fireball and a loud sonic boom, and over two metric tons of material were ultimately recovered, making it the largest carbonaceous chondrite fall ever recorded. Activity concentrations for $^{60}$Co and $^{41}$Ca are given by Nishiizumi et al. (1991b). For $^{41}$Ca the values reach up to $\sim$1500 dpm/kg(Ca) and for $^{60}$Co the highest value is $\sim$175 dpm/kg. There is a linear correlation between $^{60}$Co and $^{41}$Ca activity concentrations (Nishiizumi et al. 1991b). The trend given by the experimental data is shown by the solid black line in Fig. 5. The results for the model calculations are shown by the solid symbols. For modeling, a Co concentration of 489 ppb (Clark et al. 1971) and a solar modulation parameter $M_{{\mathrm{CL}}}$ = 600 MV is used. The latter is a relatively good proxy for the ten years before the fall of the Allende meteorite, i.e., for the time relevant for $^{60}$Co production, but is also the long-term average relevant for $^{41}$Ca production in the meteorite orbits. The dots shown for the model calculations indicate different shielding depths; low values are for close to the pre-atmospheric surface of the meteorite and high values are for larger shielding depths. The model also predicts a linear correlation, though with a slightly steeper slope. Since the range measured for $^{41}$Ca agrees well with the range predicted by the model, the discrepancy is in $^{60}$Co. Therefore, for Allende, the model slightly underestimates $^{60}$Co production, which is exactly the opposite finding as observed for Jilin.

To summarize, the new model calculations describe $^{41}$Ca production by neutron capture in the Allende meteorite in agreement with the experimental data, though the database is very limited. In contrast, production of $^{60}$Co is underestimated by up to 50%, which is exactly opposite to what has been found for the Jilin meteorite and suggests a composition dependency.

#### Neutron capture in the lunar surface—$^{41}$Ca

Nishiizumi et al. (1997) measured a $^{41}$Ca depth profile for the Apollo 15 drill core and argued that most of the $^{41}$Ca in the lunar surface is produced by thermal neutron capture on $^{40}$Ca. In a preceding study of cosmogenic radionuclide production in meteorites and the lunar surface, Leya (2025) demonstrated that the model calculations reproduce the lunar $^{41}$Ca data within the uncertainties, thereby confirming the good quality of the model calculations for thermal neutron capture in $^{40}$Ca.

#### Neutron capture in the lunar surface—Sm and Gd isotopes

Another possibility evaluating model calculations for neutron capture reactions is via isotopic shifts in Sm and Gd isotopes, which have large neutron capture cross sections and which have been used to estimate the ratio of the thermal neutron flux to the epithermal neutron flux in the lunar surface and in some types of meteorites (e.g., Hidaka et al. 2009; Hidaka and Yoneda 2009; Hidaka et al. 2000).

**Fig. 6:**
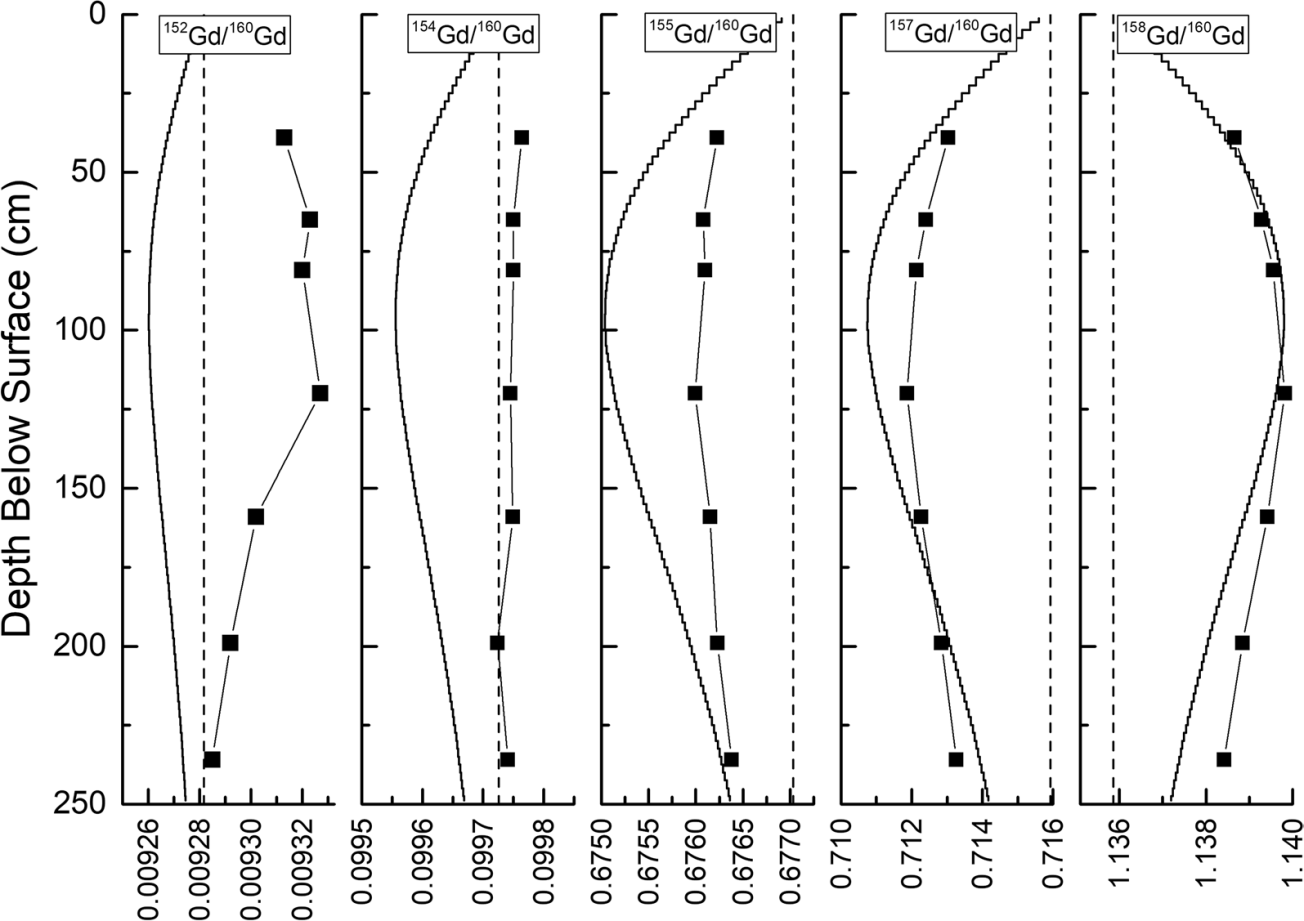
Depth profiles of (from left to right) $^{152}$Gd/$^{160}$Gd, $^{154}$Gd/$^{160}$Gd, $^{155}$Gd/$^{160}$Gd, $^{157}$Gd/$^{160}$Gd, and $^{158}$Gd/$^{160}$Gd. The depth is given in cm below the surface. The experimental data (Hidaka et al. 2000) are shown as black symbols. The results of the model calculations are shown by the solid black lines. The values for terrestrial standards, i.e., the values without any neutron capture effects, are shown by the dashed vertical lines

Figure 6 depicts depth profiles of $^{152}$Gd/$^{160}$Gd, $^{154}$Gd/$^{160}$Gd, $^{155}$Gd/$^{160}$Gd $^{157}$Gd/$^{160}$Gd, and $^{158}$Gd/$^{160}$Gd determined in the Apollo 15 drill core, after correction for mass fractionation using $^{156}$Gd/$^{160}$Gd = 0.9361. The experimental data are from Hidaka et al. (2000). The purpose of this diagram is to compare the shape of measured and modeled depth profiles. Since the magnitude of the isotopic shift depends on the irradiation time, which is not a priori known, the absolute values can be shifted toward higher or lower ratios depending on the irradiation time. For the modeled results, an irradiation time of 435 Ma is assumed. The dashed vertical lines represent the terrestrial Gd isotope ratios, i.e, the ratios before any effects due to neutron capture.

For $^{155}$Gd/$^{160}$Gd, $^{157}$Gd/$^{160}$Gd, and $^{158}$Gd/$^{160}$Gd, the trend seen in the experimental data is confirmed by the model calculations. By way of example, the $^{155}$Gd/$^{160}$Gd ratio is decreasing from the surface toward larger depths and is reaching minimum values at a depth of $\sim$100 cm. This trend can be understood considering the thermal neutron capture cross sections and resonance integrals for $^{155}$Gd ($\sigma _{{\mathrm{thermal}}}$ = 60890 barn, $I_{{\mathrm{Resonance}}}$ = 1540 barn) and $^{160}$Gd ($\sigma _{{\mathrm{thermal}}}$ = 1.41 barn, $I_{{\mathrm{Resonance}}}$ = 8.2 barn). According to these data, there is essentially no neutron capture on $^{160}$Gd but significant neutron capture on $^{155}$Gd, which produces a net burnout of $^{155}$Gd relative to $^{160}$Gd and therefore a decreasing ratio. The model and the experimental data indicate that the flux densities for the relevant neutrons are highest at a depth of $\sim$1 m. The same type of discussion holds for the $^{157}$Gd/$^{160}$Gd ratio. In contrast, since the neutron capture rates for $^{158}$Gd are low ($\sigma _{{\mathrm{thermal}}}$ = 2.202 barn, $I_{{\mathrm{Resonance}}}$ = 68.25 barn), there is no significant burnout of $^{158}$Gd but there is significant production via neutron capture on $^{157}$Gd. Therefore, the $^{158}$Gd/$^{160}$Gd ratio increases with depth; the maximum effect is at $\sim$1 m depth.

There is a discrepancy between experimental and the modeled data for $^{152}$Gd/$^{160}$Gd; while the model predicts a decreasing ratio with depth, the experimental data rather increase. Considering the thermal neutron capture cross section and the resonance integral for $^{152}$Gd of 735 barn and 558 barn, respectively, burnout of $^{152}$Gd relative to $^{160}$Gd is expected. This is seen in the model predictions, but the experimental data show the opposite trend. As an explanation, Hidaka et al. (2000) argued that the wrong trend seen in the experimental data is likely due to contributions from the reaction $^{151}$Eu(n,$\gamma \beta ^{-}$)$^{152}$Gd. A very similar argument holds for the $^{154}$Gd/$^{160}$Gd ratios, which must decrease according to the model and the capture cross sections, i.e., burnout of $^{154}$Gd, but increases according to the experimental data. In this case, contributions from the reaction $^{153}$Eu(n,$\gamma \beta ^{-}$)$^{154}$Gd might be responsible for the wrong trend (Hidaka et al. 2000). Consequently, ignoring the compromised experimental data, the model describes neutron capture effects for Gd isotopes in the lunar surface qualitatively right.

**Fig. 7:**
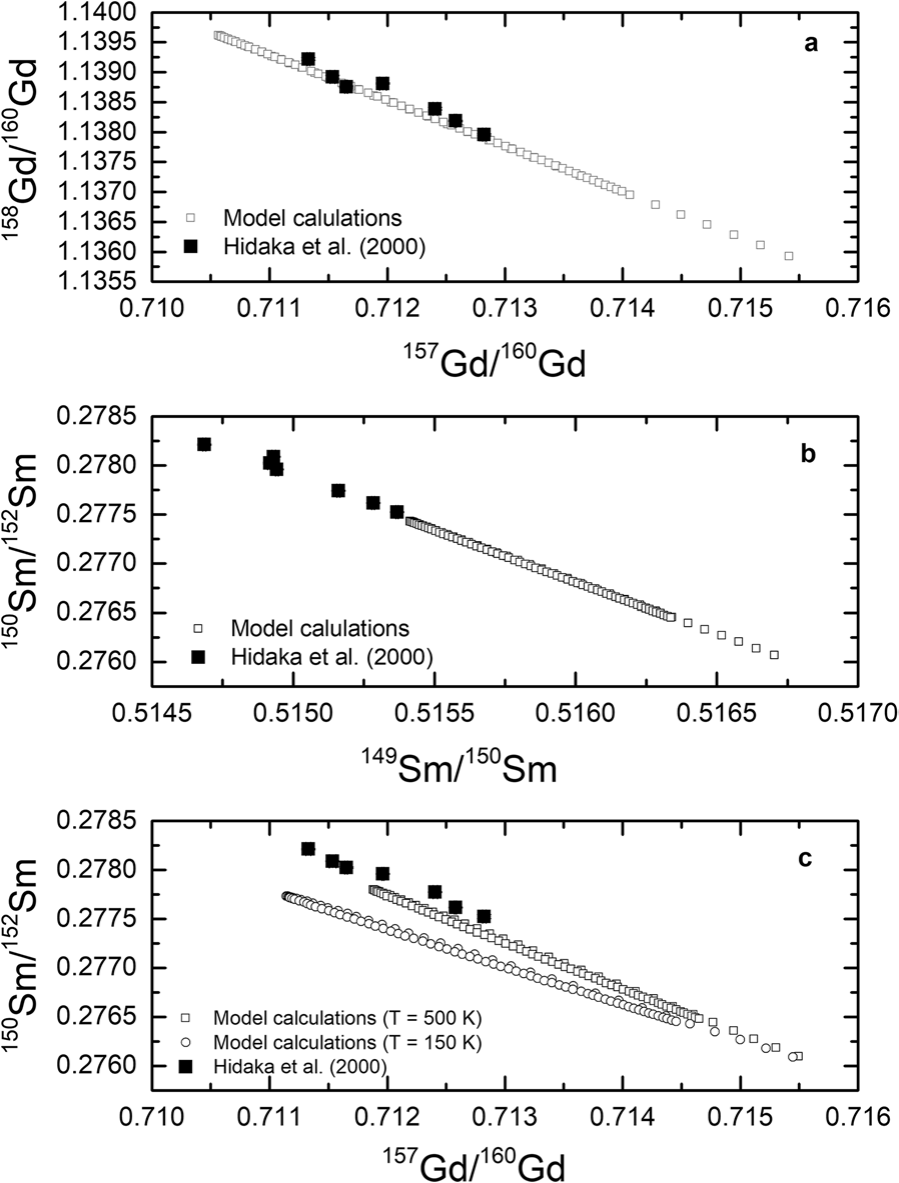
Correlated Sm and Gd isotope ratios for Apollo 15 drill core samples. $^{158}$Gd/$^{160}$Gd as a function of $^{157}$Gd/$^{160}$Gd for Apollo 15 drill core samples (panel a). $^{150}$Sm/$^{152}$Sm as a function of $^{149}$Sm/$^{150}$Sm (panel b). $^{150}$Sm/$^{152}$Sm as a function of $^{157}$Gd/$^{160}$Gd (panel c). The experimental data from Hidaka et al. (2000) are shown by the solid black symbols. The results of the model calculations are shown by the open symbols. The different symbols indicate different depths below the surface. The model calculations shown in panel c are for two different temperatures, 150 K (open dots) and 500 K (open squares)

Another way of comparing measured and modeled data is via correlated isotope effects. Fig. 7a compares measured (Hidaka et al. 2000) and modeled $^{158}$Gd/$^{160}$Gd as a function of $^{157}$Gd/$^{160}$Gd for the Apollo 15 drill core samples. There is the expected linear trend because neutron capture on $^{157}$Gd produces $^{158}$Gd and there is little neutron capture on either $^{158}$Gd or $^{160}$Gd. Therefore, the burnout of $^{157}$Gd is directly related to the production of $^{158}$Gd. The modeled trend agrees with the measured data, i.e., both are showing the same slope. Figure 7b depicts measured (Hidaka et al. 2000) and modeled $^{150}$Sm/$^{152}$Sm as a function of $^{149}$Sm/$^{152}$Sm for the same Apollo 15 samples. Again, there is a linear trend caused by neutron capture on $^{149}$Sm to produce $^{150}$Sm. This trend is predicted by the model and is confirmed by the data. The correlations are relatively robust, there is no dependence on the temperature (at least not for temperatures in the range 100–500 K). For modeling, an irradiation time of 425 Ma is assumed. For the model predictions, a longer irradiation time would extend the database toward the upper left, i.e., the data would move toward higher $^{158}$Gd/$^{160}$Gd ($^{150}$Sm/$^{152}$Sm) and lower $^{157}$Gd/$^{160}$Gd ($^{149}$Sm/$^{152}$Sm) ratios. The modeled trend for Gd overlaps with the range given by the experimental data. In contrast, the model falls slightly short describing the measured Sm isotope data (for the same irradiation time). Consequently, there is some inconsistency, While the model agrees with the isotope shifts for Gd isotopes, it slightly underestimates the shifts for Sm isotopes (for the same settings).

This discrepancy is demonstrated in Fig. 7c, where $^{150}$Sm/$^{152}$Sm is plotted as a function of $^{157}$Gd/$^{160}$Gd. The model calculations for a temperature of 500 K are shown by the open squares. There is some overlap between measured and modeled $^{157}$Gd/$^{160}$Gd data. For $^{150}$Sm/$^{152}$Sm, the same model produces too low ratios, which is simply another visualization of the finding already discussed before. However, changing the temperature of the irradiated body has an effect on the modeled results. For instance, Fig. 7c also shows the modeled ratios for a 150 K irradiation (open dots). While the model calculations are only exemplary, they clearly show the trend of decreasing $^{150}$Sm/$^{152}$Sm and increasing $^{157}$Gd/$^{160}$Gd with colder irradiation temperature. This trend is expected, considering that the major capture resonance for $^{157}$Gd is at 0.0314 eV ($\widehat{=}$ 364 K) and the major capture resonance for $^{149}$Sm is at 0.0973 eV ($\widehat{=}$ 1129 K), i.e., at a higher energy. Therefore, by reducing the temperature of the target, there are more neutrons in the very low energy range relevant for capture on $^{157}$Gd and less neutrons in the slightly higher energy range relevant for capture on $^{149}$Sm. This finding clearly demonstrates that the temperature of the target is a relevant parameter for precise neutron capture studies.

## Discussion

The discussion of $^{41}$Ca and $^{60}$Co in the two meteorites Allende and Jilin and of $^{41}$Ca and Sm and Gd isotopes in the lunar surface clearly indicates that some capture rates agree with the experimental data but that for some isotopes discrepancies exist. This section introduces the so-called response function, which helps to better understand the discrepancies and also gives a discussion on whether self-shielding might be a possible explanation.

### The differential production rates: testing the shape of the neutron spectra

**Fig. 8:**
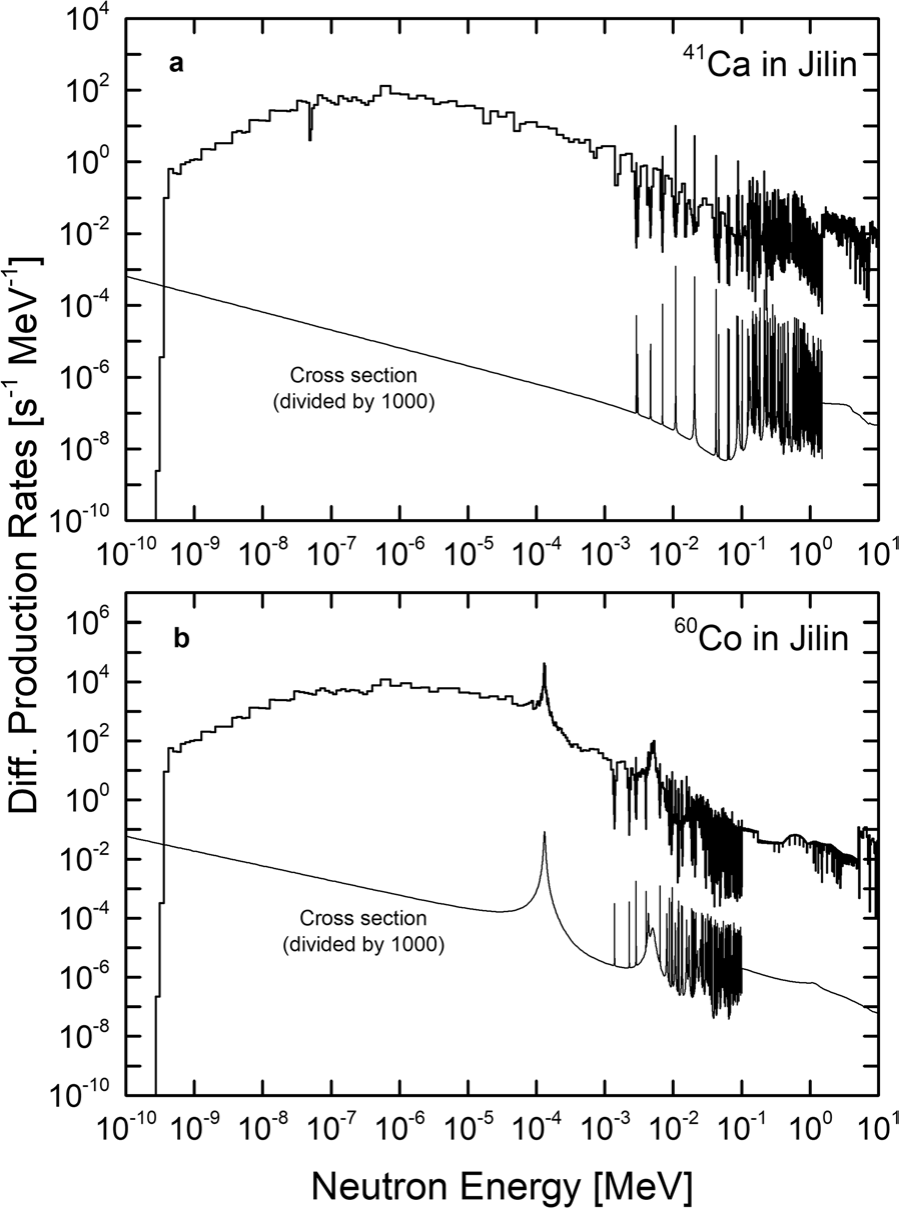
Response function (s$^{-1}$MeV$^{-1}$) for the production of $^{41}$Ca (**a**) and $^{60}$Co (**b**) for the H-chondrite Jilin. The response function for $^{41}$Ca shows a broad maximum from 10$^{-10}$ MeV to 10$^{-2}$ MeV, indicating that neutron capture is over a wide range of energies. The response function for $^{60}$Co shows a pronounced peak at $\sim$10$^{-4}$ MeV, i.e., at the energy of the major capture resonance in $^{59}$Co

To better understand the discrepant results for $^{41}$Ca and $^{60}$Co, Fig. 8 depicts the differential production rates for both isotopes at the center of the Jilin meteorite. Differential production rates $P_{diff}(E)$ at energy *E* are defined as:

6 $$\begin{aligned} P_{{\mathrm{diff}}}(E) = \phi (E) \times \sigma (E) \,\, \left[ \mathrm{MeV}^{-1} \mathrm{s}^{-1} \right] \end{aligned}$$

The parameters $\phi (i)$ $\left[ \mathrm{cm}^{-2} \mathrm{MeV}^{-1} \mathrm{s}^{-1} \right]$ and $\sigma (i)$ $\left[ \mathrm{cm}^{2} \right]$ are the neutron flux density and the cross section, respectively. The integral of the differential production rates is, aside from some factors for normalization like the element concentrations, equivalent to the capture rates. The differential production rates indicate which energy range is the most important for the capture rate, i.e., which energy range contributes most to the integral. Furthermore, for comparative purposes, the cross sections of the respective reactions are also shown, scaled by 1/1000 to enhance visual separation. Figure 8a depicts the differential production rates for the neutron capture production of $^{41}$Ca from $^{40}$Ca. The data show a broad maximum of production in the energy range 10$^{-8}$–10$^{-4}$ MeV. There is no significant peak, which is due to the fact, that the neutron capture cross section for $^{40}$Ca has no significant resonance and the missing structure in the capture cross section is directly reflected in the missing structure in the differential production rates. The production of $^{60}$Co shows a different trend. There is the same broad peak as for $^{40}$Ca but most $^{60}$Co is produced in a relatively narrow energy range slightly above 10$^{-4}$ MeV, which is at the same energy as the main capture resonance (Fig. 8b). Therefore, while most of the $^{60}$Co is produced via neutron capture at the major resonance, the majority of $^{41}$Ca is produced in a wide range of energies due to the structureless $^{40}$Ca neutron capture cross section, which mostly follow the expected 1/*v* behavior, with *v* the neutron velocity.

The reason for the finding that the model accurately describes $^{41}$Ca but under- and overestimates $^{60}$Co might be due to the fact that minor errors in modeling neutron moderation are less consequential for $^{41}$Ca production than for $^{60}$Co production. If the neutron spectra in the energy range of 10$^{-8}$–10$^{-4}$ MeV are not well described by GEANT4, the $^{59}$Co capture rates can significantly be off but the $^{40}$Ca capture rates can still be fine.

In the case of Jilin, the too high capture rate for $^{59}$Co indicates that either too many high energy neutrons are moderated down into the energy bin close to the resonance energy ($E_{r}\sim 10^{-4}$ MeV) or that too few neutrons are moderated down from this energy range toward even lower energies. In any case, there are too many (60% in the case of Jilin) neutrons with energies in the range of 10$^{-4}$ MeV. In this picture, such discrepancies affect the neutron capture on $^{40}$Ca much less because neutrons missing in one energy range are in excess in another energy range and as long as this effect is in the energy range relevant for $^{41}$Ca production, it almost cancels out. Note that the $^{60}$Co resonance is at a much higher energy than the resonance energies discussed above for Sm and Gd isotopes.

### Self-shielding of neutrons

Self-shielding refers to the reduction in the (thermal) neutron flux within a material due to its own absorption properties. Since neutrons are absorbed in one part of the target, they cannot reach other parts, which are therefore effectively shielded. For the case of $^{60}$Co in Jilin, neutron absorption at the large resonance in $^{59}$Co reduces the neutron flux in this specific energy range, thereby reducing the real capture rates. The self-shielding factor *G* quantifies the reduction in the neutron flux due to self-absorption. It is defined via:

7 $$G = \frac{{{\text{Effective average flux inside the meteorite}}}}{\text {Flux without self-shielding}} = \frac{\text {actual reaction rate}}{\text {reaction rate assuming homogeneous flux}}$$

The flux at a given position inside a target depends via the Beer-Lambert law on the macroscopic cross section $\Sigma _{a}$), which is defined as:

8 $$\begin{aligned} \Sigma _{a} = N_{i} \times \sigma _{i} \,\, \left[ \mathrm{cm}^{-1}\right] \end{aligned}$$

where $\sigma _i$ is the thermal neutron absorption cross section and $N_{i}$ is the particle density. For example, the macroscopic cross section for Jilin bulk material is 0.749 cm$^{-1}$ ($=\Sigma _{a}$), without Co it is 0.646 cm$^{-1}$ ($=\Sigma _{a}^{*}$). The mean free path of thermal neutrons, i.e., the inverse of the macroscopic cross section, for Jilin bulk material is 1.34 cm, without Co it would be 1.54 cm. Using the Beer-Lambert law, the self-shielding factor can be determined via:

9 $$\begin{aligned} \ln (G) = \ln \left( \frac{\Phi (\mathrm{average})}{\Phi (\text {without self absorption})} \right) = x \left( \Sigma _{a}^{*} - \Sigma _{a} \right) \end{aligned}$$

where *x* is a typical distance. Assuming now the mean free path as a typical distance, gives a *G* value of $\sim$0.87, i.e., $^{60}$Co self-shielding reduces the neutron flux by $\sim$13%. However, assuming as a typical distance the interaction length of GCR particles in meteoritic matter, which is $\sim$30 cm, gives $G = 4.5\times 10^{-2}$, i.e., a significant reduction of the thermal neutron flux. Note that 30 cm also corresponds to typical meteorite sizes at which neutron capture effects become relevant. This short discussion indicates that self-shielding might be of importance for nuclides with very sharp and very large resonances. For comparison, Ca, despite its much higher concentrations in Jilin, contributes less to the macroscopic cross section and therefore less to self-shielding.

As an important note, the target element Fe contributes most to the macroscopic cross section ($\Sigma _{a}$(Fe) = 0.313 cm$^{-1}$) and therefore is an important target element for neutron absorption and self-shielding. The isotope $^{56}$Fe has a relatively large neutron capture resonance of $\sim$17 barn at 10$^{-3}$ MeV and likely influences the shape of the neutron spectrum, i.e., $^{56}$Fe self-shielding might reduce the neutron flux at lower energies because some neutrons are absorbed before being moderated to lower energies and/or before being thermalized. Since the resonance for neutron capture on $^{59}$Co is at 10$^{-4}$ MeV, ignoring $^{56}$Fe self-shielding might result in too high neutron fluxes at energies below 10$^{-3}$ MeV and therefore too high neutron capture rates on $^{59}$Co to produce $^{60}$Co. While this is seen in some of the data, e.g., Jilin, the opposite trend is seen for Allende. Further tests to prove or reject this hypothesis are needed.

## Conclusions

This study investigates the capabilities of the GEANT4 Monte Carlo toolkit (Agostinelli et al. 2003; Allison et al. 2016) to quantitatively predict neutron production and neutron transport in cosmochemical relevant objects. In addition, nuclide production by neutron capture reactions is also studied.

*First*, by comparing the model predictions to experimental data for the LNPE experiment, i.e., the experiment measuring the neutron density in the lunar surface, GEANT4 with the physical list FTFP$_{-}$INCLXX$_{-}$HPT and the solar modulation parameter ($M_{{\mathrm{CL}}}$ = 438 MeV) describes the measured data within the uncertainties. This is a major improvement compared to earlier studies by Mesick et al. (2018) using an earlier version of GEANT4, McKinney et al. (2006) using MCNPX, and Ota et al. (2011) using PHITS.

*Second*, for most geo- and cosmochemical applications, production of radioactive and/or stable cosmogenic nuclides by neutron capture is more important than neutron densities. For example, neutron capture effects can be used to study the history of asteroidal and lunar regolith, the water content in such surfaces (via correlated capture rates), and very often they simply need to be understood to correct various radioactive dating systems for unwanted cosmogenic shifts. Modeling such neutron capture effects requires a precise knowledge of the neutron capture cross sections, which are available in various databases, and the neutron spectra inside the studied object, which are typically calculated using codes like GEANT4. The code used here describes experimental $^{41}$Ca activity concentrations, produced via neutron capture on $^{40}$Ca, in different types of meteorites and the lunar surface within the uncertainties. In contrast, the model has some difficulties describing $^{60}$Co activity concentrations. Whereas the modeled data are $\sim$60% higher than measured data for the large H-chondrite Jilin, they are lower compared to measured data for the carbonaceous chondrite Allende. In addition, it is difficult to consistently model the isotope shifts $^{157}$Gd/$^{160}$Gd and $^{150}$Sm/$^{152}$Sm in Apollo 15 drill core samples. If the range for $^{157}$Gd/$^{160}$Gd is described by the model, it slightly underpredicts the measured $^{150}$Sm/$^{152}$Sm ratios. This trend depends on the temperature of the irradiated object.

Since the observed discrepancies are likely related to the shape of the neutron spectra, self-shielding effects might be of importance. As an example, self-shielding induced by $^{56}$Fe in the energy range $\sim$10$^{-4}$ MeV might affect the neutron spectrum and compromise a consistent modeling of neutron capture rates for some cosmogenic nuclides.

## Data Availability

The datasets generated and/or analyzed during the current study are available in the Harvard Dataverse: https://doi.org/10.7910/DVN/A9MJAB.
